# Anthropometric factors and breast cancer risk among urban and rural women in South India: a multicentric case–control study

**DOI:** 10.1038/sj.bjc.6604423

**Published:** 2008-06-10

**Authors:** A Mathew, V Gajalakshmi, B Rajan, V Kanimozhi, P Brennan, B S Mathew, P Boffetta

**Affiliations:** 1 Regional Cancer Center, Trivandrum, India; 2 Epidemiologic Research Center, Chennai, India; 3 Rai Memorial Hospital, Chennai, India; 4 International Agency for Research on Cancer, Lyon, France

**Keywords:** breast cancer risk, anthropometric factors, urban–rural

## Abstract

Breast cancer (BC) incidence in India is approximately twice as high in urban women than in rural women, among whom we investigated the role of anthropometric factors and body size. The study was conducted at the Regional Cancer Centre, Trivandrum, and in three cancer hospitals in Chennai during 2002–2005. Histologically confirmed cases (*n*=1866) and age-matched controls (*n*=1873) were selected. Anthropometric factors were measured in standard ways. Information on body size at different periods of life was obtained using pictograms. Odds ratios (OR) of BC were estimated through logistic regression modelling. Proportion of women with body mass index (BMI)>25.0 kg/m^2^, waist size >85 cm and hip size >100 cm was significantly higher among urban than rural women. Risk was increased for waist size >85 cm (pre-menopausal: OR=1.24, 95% CI: 0.96–1.62; post-menopausal: 1.61, 95% CI: 1.22–2.12) and hip size >100 cm (pre-menopausal: OR=1.47, 95% CI: 1.05–2.06; post-menopausal 2.42, 95% CI: 1.72–3.41). Large body size at age 10 (OR=1.75, 95% CI: 1.01–3.03) and increased BMI (OR=1.33, 95% CI: 1.05–1.69 for 25.0–29.9 kg/m^2^ and OR=1.56, 95% CI: 1.03–2.35 for 30+ kg/m^2^) were associated with pre-menopausal BC risk. Our data support the hypotheses that increased anthropometric factors are risk factors of BC in India.

Breast cancer (BC) is the most common malignancy in women worldwide, with generally higher incidence rates in urban populations (Parkin *et al*, 2002). In India, the incidence is approximately twice as high among urban than rural women, ranging from 25 to 30 per 100 000 women (NCRP, 2006). Cancer registry data from the rural regions of Barshi in western India (NCRP, 2006), Karunagappally and Thiruvananthapuram – the latter two in the more developed South India – have consistently shown lower incidence than in urban registries (ranging from 7 to 20 per 100 000 females) (reports from cancer registries).

Most large studies have found that women who are overweight or obese, especially those who gain weight throughout adulthood, are at an increased risk of BC after menopause (Friedenreich, 2002; Vainio and Bianchini, 2002; Carmichael and Bates, 2004). Conversely, in most but not all case–control and cohort studies, an inverse relationship has been found between weight and BC among pre-menopausal women (Friedenreich, 2002; Carmichael and Bates, 2004). Risk increases with increasing height (Friedenreich, 2002), whereas a positive association with waist circumference or waist-to-hip ratio (WHR) has been reported in both pre- and post-menopausal women (Connolly *et al*, 2002).

Increased body mass index (BMI) and WHR are increasingly a concern in many low-resource countries, and particularly in urban India. Two large cross-sectional studies in North India have reported that increased BMI is more common in urban women than in rural women (Chadha *et al*, 1997; Singh *et al*, 1997); this, along with other factors that vary according to residence (e.g., reproductive and other hormonal factors, diet and physical activity), may therefore contribute to the urban–rural BC differences.

The present study investigated the pattern of anthropometric factors among urban and rural women and their role in BC aetiology in India as well as their contribution to the urban–rural differences in BC rates.

## Materials and methods

In 2002–2005, a case–control study was conducted at the Regional Cancer Centre (RCC), Trivandrum, Kerala, and in three cancer hospitals in Chennai, Tamil Nadu, India. The cases (*n*=1866) were women with histologically confirmed incidence of primary BC who attended the study hospitals. The controls (*n*=1873) were women without cancer who accompanied cancer patients (those accompanying BC patients were excluded), and who matched to cases by age (±5 years) and residence status (urban/rural). The institutional review boards of each participating centre approved the study. Written informed consent was obtained from all participants. Participation rates were more than 90% for both cases and controls.

In-person interview of each case and control was conducted by trained interviewers using a pre-tested structured questionnaire covering demographic and socioeconomic variables, reproductive history, time spent in household activities on a normal day, residential history, occupational history, personal and family medical history, tobacco and alcohol habits, and diet. Anthropometric measurements were taken at the end of the interview.

All subjects were asked to list all places of residence where they had lived for at least 1 year, starting with the place of birth. Urban/rural residence status was collected according to the definition of national census. If the subject lived in a ‘panchayat’, residence status was defined as ‘rural’ and all other areas such as ‘municipality’ and ‘corporation’ as ‘urban’. If the subject migrated to an urban area and lived there during the immediate previous 10 years, residential status was assigned as ‘urban’ and vice versa.

Socioeconomic status was assessed by summing the independent scores given to home ownership, number of rooms, number of people living in the house, availability of toilet and running water as well as possession of comfort/luxury items, such as electrical/gas stove, refrigerator, TV, air conditioner, car, motorcycle/scooter, bicycle and computer, owned by the subject.

**Figure 1:**
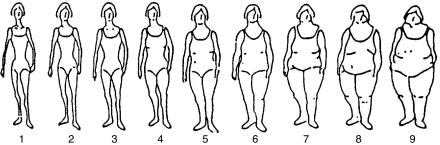
Body size.

Height (without shoes in cm) and weight in light clothing (in kg) of each subject were measured using standard equipment. Weight was measured with light clothing. Waist size (in cm) was measured using a tape at the navel level around the skin, and hip size (in cm) was measured with light clothing at the widest part. All measurements were done twice in succession and averaged for a final value. Body mass index (kg/m^2^) was grouped into three categories, namely lean weight (BMI⩽25), overweight (25<BMI<30) and obese (BMI⩾30) (WHO, 1998). Waist-to-hip ratio was computed by taking the ratio of waist size (in cm) and hip size (in cm) and grouped into two categories, namely ⩽0.85 and >0.85 (Royal College of Physicians Report, 1998). Furthermore, a total of nine different body sizes (pictogram) (Figure 1) were shown to each subject to indicate their body sizes at different periods of life (at 10 years, 20 years and the period when the data were collected).

### Data analysis

The distribution of various anthropometric factors among urban and rural women in the control group was obtained and the differences were tested using the *χ*^2^ statistic (Fisher's exact test was used if the expected value of a cell was less than 5 (Armitage and Berry, 1994). The odds ratios (OR) of BC and their 95% confidence intervals (CI) for anthropometric factors and body size were estimated separately by menopausal status and residence status through unconditional logistic regression models adjusted for age at recruitment, centre, religion, marital status, education, socioeconomic status, residential status, age at first childbirth, menopausal status, parity, duration of breast feeding, level of physical activity and other factors (Breslow and Day, 1980). Multiplicative terms were added to the regression models to test for the interaction between anthropometric factors and physical activity. The ORs were modelled using a linear relationship between the anthropometric factors/body size and the log odds of disease. All analyses were performed using the statistical package SPSS.

## Results

There were 1866 cases (735 urban and 1131 rural women) and 1873 controls (631 urban and 1242 rural) in the study. Approximately 64% of urban cases were from Chennai, whereas 80% of rural cases were from Trivandrum; approximately 63% of urban controls were from Chennai and 79% of rural controls were from Trivandrum. Of cases, 21 and 24% of controls in Chennai moved from rural areas to live in urban areas during the past 10 years, whereas the corresponding figures in Trivandrum were 10 and 8%. Migration from urban to rural areas, during the past 10 years and continued residence in rural areas was 9 and 7%, respectively, for cases and controls in Trivandrum and only 3 and 2%, respectively, in Chennai.

Socioeconomic status was significantly different among urban and rural women in Trivandrum (19 *vs* 11% in the highest quintile) and Chennai (33 *vs* 13% in the highest quintile). The proportion with higher education was higher among urban than rural women (15 *vs* 12% for education >12 years in Trivandrum and 6 *vs* 0.4% in Chennai). In Chennai, Christians and Muslims were more frequent in urban than in rural women (11 *vs* 6% Christians and 7 *vs* 4% for Muslims), whereas urban–rural religious proportions were similar in Trivandrum.

The prevalence of obesity in urban women was 9 and 10%, respectively, in Trivandrum and Chennai, whereas the corresponding figures among rural women were 3 and 5%. Approximately 36 and 30% of women in Trivandrum and Chennai urban areas had waist size >85 cm, whereas the corresponding proportions in the rural population were 21 and 18%. Similarly, the proportion of hip size >100 cm was higher in urban than in rural women (16 *vs* 7% in Trivandrum and 23 *vs* 14% in Chennai). No difference according to WHR between the urban and rural populations was observed. Body size at 10 years of age was higher in the urban women in both Chennai and Trivandrum, whereas body size at age 20 and at the time of interview was higher in the urban women only in Chennai (Table 1).

Among pre-menopausal women, an increased BC risk was observed for BMI>25.0 (OR=1.33 (95% CI: 1.05–1.69) for BMI: 25.0–29.9 and OR=1.56 (95% CI: 1.03–2.35) for BMI⩾30), waist size >85 cm (OR=1.24, 95% CI: 0.96–1.62), hip size >100 cm (OR=1.47; 95% CI: 1.05–2.06) and increased body size at 10 years of age (OR=1.75; 95% CI: 1.01–3.03 for figures 4–9 of the pictogram). In the stratified analysis, the corresponding risks were slightly higher among pre-menopausal rural women, but none was significant among pre-menopausal urban women (Table 2).

Among post-menopausal women, an increased BC risk was observed for height ⩾160 cm (OR=1.61; 95% CI: 1.08–2.42), waist size >85 cm (OR=1.61; 95% CI: 1.22–2.12) and hip size >100 cm (OR=2.42; 95% CI: 1.72–3.41), increased body size at 20 years (OR=1.23; 95% CI: 0.90–1.70) and the body size at the time of interview (OR=1.29; 95% CI: 0.92–1.90). In the stratified analysis, similar BC risks were observed among pre-menopausal urban and rural women (Table 2). None of the terms of interaction between anthropometric factors and levels of physical activity was statistically significant (data not shown).

## Discussion

The present study is of the urban–rural differences in BC incidence centres on anthropometric factors and body size at different time periods of life and their relationship to BC risk. The proportion of women with augmented anthropometric factors and larger body size in their early years of life was higher among urban women, in accord with two cross-sectional studies in North India comprising several thousand individuals (Chadha *et al*, 1997; Singh *et al*, 1997). Although the present study was hospital-based in design, the BMI assessed among controls in urban and rural areas was in agreement with the above-mentioned studies in North India, indicating their representative nature for the population.

Several anthropometric factors were associated with BC risk in both pre- and post-menopausal women and in both urban and rural women. Although these factors appear to contribute to BC aetiology in India, they are unlikely to explain most of the urban–rural difference in BC rates in India. For example, assuming that the factors associated with increased OR are true and that the exposure in controls shown in Table 3 is representative, hip size >100 cm would explain 12% of BC in urban and 9% in rural women or 9% and 11%, respectively, for waist size >85 cm. On the other hand, greater opportunities for diagnosis in urban areas may contribute to some extent.

Few studies have investigated anthropometric factors and BC risk in India. Our findings of increases associated with augmented anthropometric factors in post-menopausal women accord with previous results (Friedenreich, 2002; Vainio and Bianchini, 2002; Carmichael and Bates, 2004). However, our observation of an increased pre-menopausal BC risk with augmented anthropometric factors and larger body size in early life contrasts with previous findings mainly in high-resource countries (Friedenreich, 2002; Carmichael and Bates, 2004).

Several biological mechanisms are hypothesised to explain how anthropometric factors influence BC risk. Obesity can increase levels of circulating endogenous sex hormones, insulin and insulin-like growth factors that all, in turn, increase risk (Vainio *et al*, 2002). The findings in post-menopausal women accord with previous studies mainly in high-resource countries (Vainio and Bianchini, 2002; Carmichael and Bates, 2004).

The lack of an association between increased WHR and BC risk is again inconsistent with previous evidence (Connolly *et al*, 2002; Harvie *et al*, 2003), perhaps reflecting measurement error and a different effect of fat distribution in India compared to that in high-resource countries. Weight gain during adulthood has been widely identified as a risk factor for post-menopausal BC (Trentham-Dietz *et al*, 2000; Han *et al*, 2006), being considered a ‘probable’ cause by the WCRF (1997). We found that increased body size at early years of life and at the time of interview increased BC risk for both pre- and post-menopausal women.

As with any case–control study, case participants may have recalled certain exposures differently from controls, especially for exposures widely thought to be BC-associated. In fact, the relationship with body size was largely unknown to our subjects, thus this source of bias is unlikely. However, measurement error (non-differential misclassification), leading to the loss of power and underestimation of OR, is a plausible source of bias. Another potential bias is that control women (accompanying the cancer patients) were chosen because they were more mobile (and consequently less obese). However, the anthropometric factors were measured in most case women after their primary surgery, with some weight loss, and thus the BC risk might not be affected.

The 10-year arbitrarily chosen period for migration need not imply a change of lifestyle. The proportion of migrants from rural to urban areas was higher in both Trivandrum and (especially) Chennai than that from urban to rural areas. The urban–rural difference is minimal in Kerala and a typical rural lifestyle might be confined to Tamil Nadu. As low risk (rural) migrants were considered as urban, some urban–rural differences according to the various factors may have been reduced. However, the proportion of migrants from rural to urban areas and vice versa was similar among both cases and controls so that risk values might not be much affected.

Despite the limitations inherent in case–control studies, the advantages of the present study include a large size (it is the largest case–control study of BC in India), detailed assessment of anthropometric factors, large heterogeneity of exposures and more than 90% participation.

In summary, we observed that urban women were more obese and had relatively larger body size in the early years of life. A positive association was observed between BC risk and augmented anthropometric factors for both pre- and post-menopausal BC among rural and urban women. The study supports the hypotheses that increased anthropometric measures are important determinants of BC in India, although they do not appear to contribute appreciably to the urban–rural BC differences.

## Figures and Tables

**Table 1:** Distribution of controls with respect to anthropometric factors and body size

	**Trivandrum**					**Chennai**				
	**Urban (*n*=233)**		**Rural (*n*=975)**			**Urban (*n*=384)**		**Rural (*n*=281)**		
**Factors**	***N***	**%**	***N***	**%**	***P*-value**	***N***	**%**	***N***	**%**	***P*-value**
*BMI (kg/m*^*2*^)										
<25.0	136	58.4	721	73.9	0.0001	229	57.5	209	78.3	0.00001
25.0–29.9	77	33.0	220	22.6		118	29.6	38	14.2	
⩾30.0	20	8.6	34	3.5		40	10.1	13	4.9	
Unknown						11	2.8	7	2.6	
										
*Height (in cm)*										
<160	204	87.6	852	87.4	0.944	342	85.4	227	85.0	0.915
⩾160	29	12.4	123	12.6		45	11.3	33	12.7	
Unknown						11	2.8	7	2.6	
										
*Waist size (in cm)*										
⩽85	150	64.4	772	79.2	0.0001	270	67.8	212	79.4	0.002
>85	83	35.6	203	20.8		119	29.9	48	18.0	
Unknown						9	2.3	7	2.6	
										
*Hip size (in cm)*										
⩽100	197	84.5	902	92.5	0.0001	299	75.1	223	83.5	0.019
>100	36	15.5	73	7.5		90	22.6	37	13.9	
Unknown						9	2.3	7	2.6	
										
*WHR*										
⩽0.85	53	22.7	260	26.7	0.22	150	37.7	103	38.6	0.657
>0.85	180	77.3	715	73.3		248	62.3	157	58.8	
Unknown								7	2.6	
										
*Body size at 10 years* a										
Figure 1	95	40.8	449	46.1	0.02	87	21.9	95	35.6	0.001
Figure 2	113	48.5	450	46.2		248	62.3	146	54.7	
Figure 3	22	9.4	47	4.8		50	12.6	20	7.5	
Figures 4–9	3	1.3	29	3.0		13	3.3	6	2.2	
										
*Body size at 20 years* a										
Figures 1+2	87	37.3	362	37.1	0.79	84	21.1	89	33.3	0.001
Figure 3	112	48.1	486	49.8		187	47.0	121	45.3	
Figures 4–9 Unknown	34	14.6	127	13.0		127	31.9	57	21.3	
										
*Current body size* a										
Figures 1+2+3	9	3.9	37	3.8	0.54	188	47.2	149	55.8	0.0001
Figures 4–9	224	96.1	938	96.2		210	52.8	118	44.2	

BMI=body mass index; WHR=waist-to-hip ratio.

a See Figure 1 (pitcogram).

**Table 2:** OR of BC for anthropometric factors

	**Pre-menopausal**		**Post-menopausal**	
**Factors**	**Case/control (898/1182)**	**OR (95% CI)**	**Case/control (968/691)**	**OR (95% CI)**
*Height (in cm)* a				
<160	734/991	1.00 —	829/634	1.00 —
⩾160	147/182	1.05 (0.81–1.38)	103/48	1.61 (1.08–2.42)
Unknown	17/9	1.39 (0.41–4.76)	36/9	1.04 (0.32–3.35)
				
*Height (in cm) (urban)* b				
<160	251/294	1.00 —	341/252	1.00 —
⩾160	52/54	1.03 (0.62–1.69)	44/20	1.89 (0.97–3.67)
Unknown	13/7	1.26 (0.22–7.24)	34/4	1.45 (0.33–6.36)
				
*Height (in cm) (rural)* c				
<160	483/697	1.00 —	488/382	1.00 —
⩾160	95/128	1.05 (0.76–1.46)	59/28	1.52 (0.90–2.57)
Unknown	4/2	2.37 (0.34–16.61)	2/5	0.41 (0.03–4.94)
				
*BMI (kg/m*^*2*^)^a^				
<25	560/845	1.00 —	559/450	1.00 —
25–29.9	256/268	1.33 (1.05–1.69)	297/185	1.29 (1.00–1.66)
⩾30	65/60	1.56 (1.03–2.35)	76/47	1.00 (0.64–1.54)
Unknown	17/9	1.58 (0.46–5.42)	36/9	1.07 (0.33–3.45)
				
*BMI (kg/m* ^*2*^ *) (urban)* b				
<25	175/207	1.00 —	192/158	1.00 —
25–29.9	98/109	0.99 (0.65–1.51)	142/86	1.32 (0.89–1.97)
⩾30	30/32	1.19 (0.64–2.24)	51/28	0.89 (0.49–1.62)
Unknown	13/7	1.30 (0.26–7.56)	34/4	1.47 (0.33–6.49)
				
*BMI (kg/m* ^*2*^ *) (rural)* c				
<25	385/638	1.00 —	367/292	1.00 —
25–29.9	158/159	1.56 (1.17–2.09)	155/99	1.30 (0.92–1.83)
⩾30	35/28	1.97 (1.12–3.49)	25/19	1.15 (0.58–2.28)
Unknown	4/2		2/5	0.42 (0.03–5.16)
				
*Waist size (in cm)* b				
⩽85	631/918	1.00 —	557/486	1.00 —
>85	250/254	1.24 (0.96–1.62)	380/199	1.61 (1.22–2.12)
Unknown	17/10	1.19 (0.37–3.90)	31/6	2.88 (0.76–10.90)
				
*Waist size (in cm) (urban)* b				
⩽85	208/235	1.00 —	213/185	1.00 —
>85	96/113	0.97 (0.61–1.54)	178/89	1.71 (1.12–2.61)
Unknown	12/7	1.25 (0.20–7.83)	28/2	5.90 (0.92–37.96)
				
*Waist size (in cm) (rural)* c				
⩽85	423/683	1.00 —	344/301	1.00 —
>85	154/141	1.43 (1.03–1.99)	202/110	1.54 (1.06–2.23)
Unknown	5/13	1.34 (0.25–7.17)	3/4	1.65 (0.13–21.77)
				
*Hip size (in cm)* a				
⩽100	723/1037	1.00 —	673/584	1.00 —
>100	157/135	1.47 (1.05–2.06)	264/101	2.42 (1.72–3.41)
Unknown	18/10	1.47 (0.47–4.60)	31/6	3.46 (0.89–13.35)
				
*Hip size (in cm) (urban)* b				
⩽100	230/278	1.00 —	242/218	1.00 —
>100	73/70	1.49 (0.89–2.51)	149/56	2.65 (1.60–4.37)
Unknown	13/7	2.16 (0.40–11.76)	28/2	6.85 (1.04–45.06)
				
*Hip size (in cm) (rural)* c				
⩽100	493/759	1.00 —	431/366	1.00 —
>100	84/65	1.48 (0.94–2.34)	115/45	2.34 (1.44–3.82)
Unknown	5/3	1.34 (0.25–7.16)	3/4	2.03 (0.15–28.09)
				
*WHR* a				
⩽0.85	295/398	1.00 —	261/159	1.00 —
>0.85	585/774	0.92 (0.74–1.13)	676/526	0.74 (0.57–0.97)
Unknown	18/10	1.27 (0.40–3.98)	31/6	1.95 (0.51–7.48)
				
*Waist-to-hip ratio (urban)* b				
⩽0.85	109/124	1.00 —	123/70	1.00 —
>0.85	194/224	0.85 (0.58–1.26)	268/204	0.75 (0.50–1.13)
Unknown	13/7	1.60 (0.29–8.69)	28/2	4.10 (0.63–26.83)
				
*Waist-to-hip ratio (rural)* c				
⩽0.85	186/274	1.00 —	138/89	1.00 —
>0.85	391/550	0.93 (0.71–1.20)	408/322	0.71 (0.50–1.01)
Unknown	5/3	1.25 (0.23–6.76)	3/4	0.88 (0.08–12.22)
				
*Body size at 10 years* a				
Figure 1	329/490	1.00 —	329/236	1.00 —
Figure 2	453/572	1.12 (0.90–1.38)	488/385	0.82 (0.64–1.05)
Figure 3	76/88	1.13 (0.77–1.67)	110/51	1.26 (0.83–1.92)
Figures 4–9	40/32	1.75 (1.01–3.03)	41/19	1.26 (0.67–2.40)
				
*Body size at 10 years (urban)* b				
Figure 1	107/109	1.00 —	105/73	1.00 —
Figure 2	171/196	0.90 (0.59–1.37)	242/165	0.82 (0.53–1.27)
Figure 3	26/42	0.62 (0.31–1.22)	50/30	0.83 (0.43–1.60)
Figures 4–9	12/8	1.73 (0.58–5.12)	22/8	1.50 (0.53–4.23)
				
*Body size at 10 years (rural)* c				
Figure 1	222/381	1.00 —	224/163	1.00 —
Figure 2	282/376	1.20 (0.93–1.54)	246/220	0.78 (0.57–1.07)
Figure 3	50/46	1.45 (0.89–2.37)	60/21	1.91 (1.04–3.49)
Figures 4–9	28/24	1.84 (0.96–3.53)	19/11	1.14 (0.48–2.73)
				
*Body size at 20 years* a				
Figures 1+2	281/419	1.00 —	276/203	—
Figure 3	424/558	1.00 (0.80–1.25)	424/348	0.82 (0.63–1.06)
Figures 4–9	193/205	1.16 (0.87–1.54)	268/140	1.23 (0.90–1.70)
				
*Body size at 20 years (urban)* b				
Figures 1+2	91/97	—	98/74	1.00 —
Figure 3	157/175	0.80 (0.51–1.24)	176/124	0.89 (0.56–1.41)
Figures 4–9	68/83	0.70 (0.41–1.20)	145/78	1.05 (0.64–1.72)
				
*Body size at 20 years (rural)* c				
Figures 1+2	190/322	—	178/129	1.00 —
Figure 3	267/383	1.06 (0.81–1.38)	248/224	0.77 (0.55–1.08)
Figures 4–9	125/122	1.42 (1.01–2.00)	123/62	1.43 (0.92–2.22)
				
*Current body size* a				
Figures 1+2+3	153/227	1.00 —	163/156	1.00 —
Figures 4–9	745/955	0.90 (0.64–1.25)	805/535	1.29 (0.92–1.90)
				
*Current body size (urban)* b				
Figures 1+2+3	82/107	1.00 —	97/90	1.00 —
Figures 4–9	234/248	0.79 (0.48–1.29)	322/186	1.55 (0.97–2.48)
				
*Current body size (rural)* c				
Figures 1+2+3	71/120	1.00 —	66/66	1.00 —
Figures 4–9	511/707	1.07 (0.66–1.73)	483/349	1.18 (0.70–1.98)

BC=breast cancer; BMI=body mass index; CI=confidence interval; OR=odds ratio; WHR=waist-to-hip ratio.

a Adjusted for age, centre, religion, marital status, education, socioeconomic status, residence status, parity, age at 1st childbirth and duration of breast feeding, physical activity and variables in the table (where appropriate).

b Only urban, adjusted for age, centre, religion, marital status, education, socioeconomic status, parity, age at 1st childbirth and duration of breast feeding, physical activity and variables in the table (where appropriate).

c Only rural, adjusted for age, centre, religion, marital status, education, socioeconomic status, parity, age at 1st childbirth and duration of breast feeding, physical activity and variables in the table (where appropriate).

**Table 3:** BC risk factors by place of residence

	**Urban**			**Rural**		
**Factors**	**Case/control (*n*=735/631)**	**OR^a^**	**95% CI**	**Case/control (*n*=1131/1242)**	**OR^a^**	**95% CI**
*Height (in cm)*						
<160	592/546	1.00	—	971/1079	1.00	—
⩾160	96/74	1.27	0.87–1.86	154/156	1.16	0.89–1.52
Unknown	47/11	1.29	0.44–3.78	6/7	0.95	0.23–3.92
						
*BMI (kg/m*^*2*^)						
<25	367/365	1.00	—	752/930	1.00	—
25–25.9	240/195	1.18	0.90–1.56	313/258	1.44	1.16–1.79
>30	81/60	0.98	0.64–1.49	60/47	1.63	1.06–2.51
Unknown	44/11	1.32	0.45–3.88	5/7	1.04	0.25–4.30
						
*Waist size (in cm)*						
⩽85	421/420	1.00	—	767/984	1.00	—
>85	274/202	1.31	0.97–1.77	356/251	1.46	1.14–1.86
Unknown	40/9	2.37	0.73–7.64	8/7	1.28	0.33–4.92
						
*Hip size (in cm)*						
⩽100	472/496	1.00	—	924/1125	1.00	—
>100	222/126	2.00	1.41–2.84	199/110	1.80	1.30–2.49
Unknown	41/9	3.25	1.04–10.18	8/7	1.32	0.34–5.10
						
*WHR ratio*						
⩽0.85	232/194	1.00	—	324/363	1.00	—
>0.85	462/428	0.81	0.62–1.07	799/872	0.86	0.70–1.06
Unknown	41/9	2.14	0.68–6.73	8/7	1.08	0.28–4.16
						
*Body size at 10 years* b						
Figure 1	212/182	1.00	—	446/544	1.00	—
Figure 2	413/361	0.86	0.64–1.14	528/596	1.03	0.85–1.25
Figure 3	76/72	0.78	0.49–1.22	110/67	1.69	1.17–2.44
Figures 4–9	34/16	1.48	0.71–3.04	47/35	1.49	0.90–2.48
						
*Body size at 20-years* b						
Figures 1+2	189/171	1.00	—	368/451	1.00	—
Figure 3	333/299	0.87	(0.64–1.18)	515/607	0.95	0.78–1.17
Figures 4–9	213/161	0.95	(0.67–1.35)	248/184	1.42	1.09–1.85
						
*Current body size* b						
Figures 1+2+3	179/197	1.00	—	137/186	1.00	—
Figures 4–9	556/434	1.17	0.84–1.62	994/1056	1.12	0.79–1.58

BC=breast cancer; BMI=body mass index; CI=confidence interval; OR=odds ratio; WHR=waist-to-hip ratio.

a OR adjusted for age, centre, religion, marital status, education, socioeconomic status, parity, age at 1st childbirth, duration of breastfeeding, menopausal status, physical activity and variables in the table (where appropriate).

b Refer Figure 1.
